# Is there any relationship between match running, technical-tactical performance, and team success in professional soccer? A longitudinal study in the first and second divisions of LaLiga

**DOI:** 10.5114/biolsport.2023.118021

**Published:** 2022-09-06

**Authors:** José M. Oliva-Lozano, Helena Martínez-Puertas, Víctor Fortes, Roberto López-Del Campo, Ricardo Resta, José M. Muyor

**Affiliations:** 1 Health Research Centre. University of Almería, Almería, Spain; 2 Department of Mathematics, University of Almería, Almería, Spain; 3 Unión Deportiva Almería, Almería, Spain; 4 Department of Competitions and Mediacoach, LaLiga, Madrid, Spain; 5 Laboratory of Kinesiology, Biomechanics and Ergonomics (KIBIOMER Lab). Research Central Services. University of Almería, Almería, Spain

**Keywords:** Match Analysis, Elite, Football, LaLiga, Load, Physical Demands

## Abstract

The aim of this study was to analyze the association between teams’ success at the end of the season and match running, and technical-tactical performance in two professional soccer leagues. Match running, and technical-tactical performance data were collected during two consecutive seasons. A Factor Analysis was conducted to reduce the number of performance variables into a fewer number of factors. The scree plot with parallel analysis revealed that five factors should be retained. Then, a multiple linear regression analysis was performed to explain which variables and factors were more associated with teams’ success at the end of the season. The main findings of this study were that factor 3, which was correlated with goals scored, possessions ending with a goal, shots on target, goals from set plays, goals from a direct free kick, offsides, and goals conceded, was the most important contributor to teams’ success (β = 0.66). In addition, this study observed a significant interaction (p = 0.001) between the second division of LaLiga and factor 2, which correlated with total distance (TD), sprinting distance (SPD), and sprinting actions (SPA) when opponent team owns the ball, tackles, shots inside the box, and fouls received. This implies that factor 2 had a different effect on the total points at the end of the season depending on the league. However, factor 2 had no effect on the first division. In conclusion, technical-tactical performance variables were usually more closely linked to the team’s success in both leagues than match running performance data. Regarding the technical-tactical performance, teams may focus on drills that promote goal situations, shooting accuracy, the total of shots performed in match play, and set pieces. However, defensive skills need to be reinforced considering the importance of goals conceded for team success in both divisions. When it comes to the match running performance, teams are encouraged to focus on offensive actions, in which they possess and run with the ball (especially at high speed) and defensive actions in which the players perform continuous and high-intensity physical efforts to prevent the opponents from scoring, avoid counterattacks, stay compact, and defend the area and goal.

## INTRODUCTION

In recent years, technological advances in the sports industry have led to the rapid development of electronic performance and tracking systems (EPTS) such as global positioning systems, local positioning systems, and optical tracking systems [1]. These EPTS have allowed sports scientists and coaching and medical departments to better understand players’ performance [1, 2]. Most professional soccer teams currently use EPTS on a daily basis, with several leagues already investing in EPTS to be used on match day for both performance and media purposes. Examples of these multi-camera tracking systems are non-invasive EPTS that collect technical-tactical activity and match running performance [3]. Consequently, practitioners have access to vast amounts of data, which may serve to accurately analyze individual and team performance and the performance of the opposing team.

However, in soccer, team success is a complex construct in which different variables such as shooting accuracy, defensive actions, or running performance may play a significant role in professional soccer matches [4–7]. For instance, a recent study in a German professional soccer league concluded that variables related to shooting accuracy, such as shots on target or goal efficiency, were significantly correlated with team success at the end of the season [8, 9]. In addition, an investigation of a Chinese professional league found that variables related to ball possession and duels were also linked to team success [10].

Furthermore, match running performance may significantly contribute to team success [5–7, 10]. For instance, previous studies found that high-ranked teams covered shorter distances without ball possession than lower-ranked teams [7, 10]. Also, it was observed that distance covered with ball possession (especially running at high speed) had a positive and significant association with the total number of points obtained at the end of the season [6, 7]. Along these lines, a recent study in LaLiga concluded that the main changes in both the first and second Spanish divisions were the increase in distance covered and the total of efforts performed at high intensity [11].

However, research on the relationship between team success and match running and technical-tactical performance in professional soccer is scarce. From a practical perspective, it is necessary to analyze which performance indicators should be taken into account, not only for team success but also for training purposes and talent identification. Therefore, the aim of this study is to analyze the association between teams’ success at the end of the season and variables related to match technical-tactical and running performance in two professional soccer leagues.

## MATERIALS AND METHODS

### Study design

This study followed a longitudinal design. Match running, and technical-tactical performance data were collected by electronic performance and tracking systems during two consecutive seasons (2017/2018 and 2018/2019) in two professional soccer leagues. In addition, the total number of points obtained at the end of each season by each team were registered to analyze its relationship with match running and technical-tactical data.

### Sample

Data were collected from professional soccer teams competing in the first and second division Spanish Professional Soccer Leagues known as LaLiga. The first division is comprised of 20 teams, while the second division is composed of 22 teams. During the season, each first division team played 38 matches (home matches = 19; away matches = 19), while in the second division, each team played 42 matches (home matches = 21; away matches = 21). This resulted in a total of 3368 match observations (1520 match observations for the first division and 1848 match observations for the second division). However, as LaLiga expelled one of the second division teams during the 2018/2019 season, their data were not included in the analysis.

### Variables

Regarding match technical-tactical performance, the following variables were collected for each team: goals scored, goals conceded, ball possession (%), tackles, duels, aerial duels, recoveries, attempted crosses, successful crosses, fouls made, fouls received, yellow cards, second yellow cards, red cards, offsides, attempted passes, successful passes, passes ending in own team’s half, passes ending in opponent team’s half, passes made in the final third, possession width, possession length, possessions ending in attacking third, possessions involving 0–2 passes, possessions involving 3–5 passes, possessions involving 6–8 passes, possessions involving more than 9 passes, possessions starting in attacking third, possessions starting in the middle third, possessions ending with a shot, possessions ending with a goal, chances created from a set play, corners, goals from corners, goals from a direct free kick, goals from a set play, shots on target, shots off target, and shots inside the box. Regarding the match running performance, the following variables were included: total distance (TD) covered (m) when the opponent team possessed the ball (m), TD covered when the own team possessed the ball (m), sprinting distance (SPD, above 21 km/h) when opponent team possessed the ball (m), SPD covered when own team possessed the ball (m), total of sprinting actions (SPA, above 21 km/h) when opponent team possessed the ball, and the total of SPA when own team possessed the ball. Furthermore, each team’s total number of points obtained at the end of each season was registered as a representative variable of team success.

### Instruments

Electronic performance and tracking systems were used to collect performance variables. Specifically, the computerized multi-camera tracking system TRACAB (ChyronHego, New York, USA) collected positioning and motion data while events were recorded by OPTA (Opta Sports, London, UK). The validity and reliability of these systems have been assessed in previous studies [12–14]. Subsequently, reports that took into account all thevariables were generated using Mediacoach software.

### Statistical analysis

**FIG. 1:**
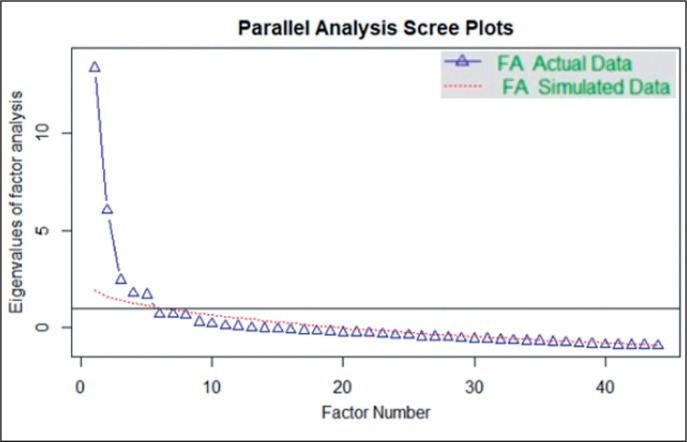
Scree plot with parallel analysis indicating that five factors should be retained in the factor analysis (FA).

First, a descriptive analysis of the match running and technical-tactical performance in both leagues was performed. Then, an Exploratory Factor Analysis was conducted to reduce the dimension of the performance variables. The appropriateness of factor analysis was determined through Kaiser Meyer Olkin measure of sampling adequacy (KMO = 0.7) and Bartlett’s test of sphericity (X^2^ = 5308.34; df = 496; *p* < 0.001). The number of factors was determined using a Scree plot with Parallel Analysis (Figure 1), which revealed that five factors should be retained (i.e., the “elbow” of the curve). The Exploratory Factor Analysis was computed through a maximum likelihood method and the interpretation of the factors was based on orthogonal rotation with the varimax method, since it is the most appropriate when the independence of the factors is assumed [15, 16].

Finally, a multiple linear regression analysis was performed to study the association between teams’ success and match performance. Homoscedasticity and linearity assumptions of the linear regression models, as well as the normality, no multicollinearity and no autocorrelation of the residuals were checked. The total of points obtained at the end of the season was set as the dependent variable while the leagues (i.e., first or second division), the score values of retained factors, and the variables, which were not well represented by the factors (i.e., low correlations with retained factors), were set as independent variables. In addition, the interactions between these independent variables and the leagues were included in the model to analyze the effect that the league had on the total of points (i.e., to analyze if the effect of any independent variable on the total of points was different depending on the league). Coefficient of determination (R^2^) was used as predictive success criteria for the regression model. All statistical analyses were carried out using R Version 3.6.1 (The R Foundation for Statistical Computing, Vienna, Austria) and the R Studio interface Version 1.1.463 (RS Team—RStudio, Inc., Boston, Massachusetts, United States). Statistical significance was set at *p* < 0.05.

## RESULTS

Table 1 shows descriptive statistics of match running and technical-tactical performance in the first and second divisions of LaLiga. An Exploratory Factor Analysis was conducted to reduce the dimension of the match technical-tactical and running performance variables and a five-factor model (F1, F2, F3, F4, and F5), which explained 61% of the common variance, was obtained. Factors loadings were estimated, and Table 2 shows the greatest correlations between the performance variables and corresponding factors. Aerial duels, possession width, second yellow cards, and red cards had low correlations with the factors. Consequently, these variables were not well represented by none of the retained factors.

**TABLE 1:** Match running and technical-tactical performance in the first and second division of LaLiga

	First Division			Second Division		

Variables	Mean ± SD	Min	Max	Mean ± SD	Min	Max
Goals scored	1.32 ± 0.43	0.63	2.61	1.14 ± 0.22	0.69	1.64
Goals conceded	1.32 ± 0.32	0.58	2.00	1.13 ± 0.27	0.68	1.93
Ball possession (%)	50.00 ± 6.32	40.10	64.40	50.01 ± 3.35	41.73	54.85
Tackles	42.14 ± 2.76	36.51	46.80	44.52 ± 2.40	38.92	49.48
Duels	50.00 ± 2.21	44.66	54.09	50.01 ± 1.35	47.42	52.94
Aerial duels	50.00 ± 3.20	41.33	55.77	50.05 ± 2.38	45.30	56.00
Recoveries	53.49 ± 2.55	47.47	60.18	52.26 ± 2.60	46.63	59.83
Attempted crosses	18.12 ± 3.74	10.37	27.82	18.69 ± 2.52	13.21	23.86
Successful crosses	4.22 ± 1.06	2.21	7.00	4.13 ± 0.69	2.60	5.88
Fouls made	13.71 ± 1.75	10.08	17.71	15.20 ± 1.30	12.45	17.22
Fouls received	13.71 ± 1.32	11.26	18.26	15.17 ± 1.02	13.48	17.59
Yellow cards	2.50 ± 0.40	1.61	3.42	2.60 ± 0.37	1.86	3.37
Second yellow cards	0.06 ± 0.04	0.00	0.13	0.08 ± 0.04	0.00	0.17
Red cards	0.04 ± 0.02	0.00	0.08	0.04 ± 0.03	0.00	0.12
Offsides	2.40 ± 0.47	1.53	3.61	2.34 ± 0.41	1.26	3.10
Attempted passes	433.35 ± 88.54	295.47	666.00	392.34 ± 42.52	279.76	478.24
Successful passes	344.43 ± 94.45	188.13	587.89	295.85 ± 46.28	173.67	395.05
Passes ending in own team’s half	190.93 ± 47.97	93.39	274.29	174.61 ± 32.52	83.74	249.66
Passes ending in opponent team’s half	242.42 ± 48.85	185.89	391.71	217.72 ± 16.95	194.54	262.02
Passes made in the final third	54.80 ± 6.70	44.37	76.58	55.42 ± 4.35	47.22	66.74
Possession length	23.34 ± 1.96	20.32	29.29	22.78 ± 1.00	20.98	25.00
Possession width	33.99 ± 0.73	32.25	35.53	34.04 ± 0.83	32.46	36.22
Possessions ending in attacking third	37.78 ± 3.72	32.66	47.26	37.80 ± 2.66	31.10	43.33
Possessions (0–2 passes)	42.50 ± 8.37	25.29	60.37	48.06 ± 5.73	38.51	64.90
Possessions (3–5 passes)	26.32 ± 2.65	20.47	31.08	28.15 ± 2.00	23.55	32.31
Possessions (6–8 passes)	12.60 ± 1.38	9.84	15.84	12.51 ± 1.27	8.86	15.10
Possessions (> 9 passes)	16.07 ± 5.94	5.58	30.87	13.09 ± 3.15	4.38	19.93
Possessions starting in attacking third	6.75 ± 1.15	5.26	11.32	6.55 ± 0.99	4.17	8.79
Possessions starting in middle third	37.42 ± 3.92	32.34	50.92	40.05 ± 3.49	32.05	47.44
Possessions ending with a shot	10.62 ± 1.47	8.74	16.18	10.20 ± 1.03	8.48	12.76
Possessions ending with a goal	1.28 ± 0.42	0.61	2.53	1.10 ± 0.22	0.64	1.60
Chances created from set play	1.98 ± 0.41	1.21	2.95	1.97 ± 0.29	1.29	2.54
Corners	4.85 ± 0.84	3.58	7.34	4.91 ± 0.54	3.81	6.17
Goals from corners	0.15 ± 0.08	0.03	0.42	0.14 ± 0.06	0.00	0.26
Goals from direct free kick	0.04 ± 0.04	0.00	0.16	0.04 ± 0.03	0.00	0.12
Goals from set play	0.27 ± 0.11	0.05	0.55	0.28 ± 0.09	0.10	0.48
Shots on target	4.24 ± 0.99	3.00	7.45	3.91 ± 0.49	3.05	4.95
Shots off target	5.09 ± 0.72	4.03	7.16	4.97 ± 0.52	4.07	6.02
Shots inside the box (%)	61.76 ± 4.31	51.81	72.93	57.91 ± 3.81	50.69	67.60
TD when opponent team owns the ball (m)	41372.72 ± 3518.80	34104.61	48213.08	38955.44 ± 1867.74	35740.56	42844.33
TD when own team owns the ball (m)	37933.37 ± 4374.40	29748.76	46029.67	36014.98 ± 2978.50	27116.51	42222.32
SPD when opponent team owns the ball (m)	3149.84 ± 295.57	2446.73	3786.68	2906.38 ± 199.08	2526.55	3347.27
SPD when own team owns the ball (m)	2652.42 ± 287.90	2203.62	3424.89	2493.61 ± 257.00	1774.76	2997.83
SPA when opponent team owns the ball	165.92 ± 15.46	136.65	199.81	151.56 ± 9.97	130.40	177.12
SPA when own team owns the ball	123.62 ± 13.98	96.73	157.95	113.84 ± 11.18	81.93	136.60

**Note:** SD = standard deviation; Min: minimum; Max: maximum; TD: total distance; SPD: sprinting distance; SPA: sprinting actions

**TABLE 2:** Factor loadings of the match technical-tactical and running performance using a maximum likelihood method and Varimax rotation

Variable	Factor 1	Factor 2	Factor 3	Factor 4	Factor 5
Possessions (> 9 passes)	0.98				
Successful passes	0.97				
TD when own team owns the ball (m)	0.94				
Possessions (0–2 passes)	-0.93				
Passes ending in own team’s half	0.91				
Ball possession (%)	0.86				
Passes ending in opponent team’s half	0.81				
Possessions (3–5 passes)	-0.78				
Fouls made	-0.73				
SPA when own team owns the ball	0.71				
Possessions (6–8 passes)	0.61				
Possessions ending with a shot	0.59				
Possession length	0.58				
Duels	0.58				
SPD when own team owns the ball (m)	0.50				
Yellow cards	-0.48				

SPA when opponent team owns the ball		0.88			
SPD when opponent team owns the ball (m)		0.85			
TD when opponent team owns the ball (m)		0.63			
Tackles		-0.51			
Fouls received		-0.40			
Shots inside the box (%)		0.39			
Possession width		-0.12			

Goals scored			0.83		
Possessions ending with a goal			0.83		
Shots on target			0.61		
Goals from set play			0.53		
Goals from direct free kick			0.39		
Offsides			0.37		
Goals conceded			-0.35		

Possessions starting in middle third				0.82	
Passes made in the final third				0.79	
Possessions starting in attacking third				0.73	
Recoveries				0.62	
Possessions ending in attacking third				0.61	
Second yellow cards				-0.27	
Aerial duels				0.23	

Attempted crosses					0.85
Successful crosses					0.84
Shots off target					0.71
Chances created from set play					0.60
Corners					0.59
Goals from corners					0.49
Red cards					0.12

**% of variance**	**26**	**7**	**9**	**9**	**10**

Specifically, Table 3 shows the multiple linear regression assessing the association between team success and match performance in the first and second divisions of LaLiga. The F-test was significant (F = 9.21; df = 19 and 63; *p* < 0.001) and the adjusted model explained 73.5% (R^2^ = 0.735) of the variance of team’s success at the end of the season.

**TABLE 3:** Multiple linear regression analysis assessing the association between team success and match performance in the first and second division of LaLiga

		B	*p*
Intercept		50.221	0.000
Second division	0.418	12.108	0.001
Aerial duels	0.113	0.591	0.313
Second yellow cards	-0.059	-20.479	0.610
Red cards	0.010	4.899	0.942
Possession width	-0.032	-0.603	0.783
F1	0.220	3.186	0.068
F2	-0.102	-1.436	0.248
F3	0.656	9.365	0.000
F4	0.136	1.938	0.166
F5	0.004	0.0627	0.976
Second division * Aerial duels	0.146	1.248	0.175
Second division * Second yellow cards	-0.048	-24.998	0.653
Second division * Red cards	0.026	16.525	0.838
Second division * Possession width	0.153	3.736	0.193
Second division * F1	0.154	4.187	0.140
Second division * F2	0.228	5.473	0.013
Second division * F3	0.091	2.554	0.301
Second division * F4	-0.091	-2.037	0.345
Second division * F5	0.060	1.641	0.631

β: standardized regression coefficient; B: non-standardized regression coefficient

F3 (goals scored: 0.83; possessions ending with a goal: 0.83; shots on target: 0.61; goals from set play: 0.53; goals from direct free kick: 0.39; offsides: 0.37; goals conceded: -0.35) was statistically significant (*p* < 0.001) and had a positive effect (B = 9.365) on the total of points obtained at the end of the season. This implies that the teams’ success was associated with increases in the value of F3 (i.e., increasing goals scored, possessions ending with a goal, shots on target, goals from set play, goals from direct free-kick and offsides whereas the value of goals conceded decreases). Furthermore, according to the Beta weights, which compared the relative importance of each independent variable or factor in standardized terms, showed that F3 had the highest impact on the teams’ success (β = 0.66).

In addition, the interaction between the second division and F2 (SPA when opponent team owns the ball: 0.88; SPD when opponent team owns the ball: 0.85; TD when opponent team owns the ball: 0.63; tackles: -0.51; fouls received: -0.40; shots inside the box: 0.39) was statistically significant (*p* = 0.001) and had a positive effect (B = 5.473) on the total of points at the end of the season. This implies that F2 had a different effect on the total points at the end of the season depending on the league (i.e., first or second division). Specifically, the teams’ success was associated with increases in the value of F2 in the second division (i.e., increasing TD, SPD, and SPA when opponent team owns the ball and shots inside the box whereas tackles and fouls received decrease) whereas this factor had no effect on team success in the first division.

## DISCUSSION

The aim of this study was to analyze the association between teams’ success (based on the total of points obtained at the end of the season) and match performance in two professional soccer leagues. The main findings of this study were that F3, which correlated with goals scored, possessions ending with a goal, shots on target, goals from a set play, goals from a direct free kick, goals conceded, and offsides, was the most important contributor to teams’ success in both divisions. In addition, this study observed a significant interaction between the second division of LaLiga and F2, which correlated with TD, SPD, and SPA when opponent team owns the ball, tackles, shots inside the box, and fouls received. However, F2 had no significant effect on team success in the first division

One of the main findings, consistent with previous research [7, 10, 17], was that technical-tactical performance variables were usually more closely related to the team’s success than match running performance. The results show that not only goals scored determine team success but also how much teams generate close to the opponent’s goal (e.g., shots on target or set pieces) and the goals conceded. These may be expected results considering the nature of soccer and previous investigations, which suggested that higher-ranked teams had better technical-tactical performance than lower-ranked teams in both offense- and defense-related variables [7, 8, 18–20]. For example, a recent study in the German Bundesliga [7] showed significant correlations between the total of points obtained at the end of the season and both offense-related (e.g., goals scored: r = 0.90; assists: r = 0.88; successful passes from open play: r = 0.78; or ball possession ratio: r = 0.78) and defense-related variables (e.g., allowed shots on goal: r = -0.87; goals conceded: r = -0.81; or saved shots on goal by the goalkeeper: r = 0.70). Moreover, a systematic review investigating how to be successful in soccer concluded that the most influential technical-tactical variables were goals per shot, shots on goal, possession, and successful passes [21]. However, team success is also dependent on contextual variables such as quality of opponent and match location, which may play an important role as well [21].

Another novel finding of this study was that a significant interaction between the second division of LaLiga and F2 was observed. This factor, which correlated with match running performance variables (i.e., TD, SPD, and SPA when the opponent team owns the ball) and technical-tactical variables (i.e., fouls received, tackles, and shots inside the box) was a significant contributor to team success in the second division of LaLiga. This may be due to the effect of situational variables such as scoreline. For example, winner teams may spend more time preventing the opponents from scoring, which may imply greater physical efforts to avoid counterattacks, stay compact, and defend the area and goal. However, previous studies observed significant negative associations between team success and distance covered without ball possession in professional soccer leagues [5, 6]. In addition, our results showed that TD, SPD, and SPA with ball possession had a positive correlation with F1, which was significantly correlated with team success at the 0.1 level (*p* = 0.07). Specifically, F1 was mainly associated with variables related to ball possession, which suggests that higher-ranked teams have greater tactical awareness or technical ability to possess and run with the ball [22]. The above notwithstanding, match running performance seems to play an essential role in team success, especially in current soccer matches, demanding greater distance covered a and total number of actions performed at high intensity [11, 23].

This study presents some limitations which need to be acknowledged. For instance, no internal load variables (e.g., mean heart rate) were collected. Also, additional external load variables (e.g., a total of accelerations or decelerations) could be included in future studies. Also, the influence of different contextual variables (e.g., length of the microcycle, match location, or scoreline) on match performance were not considered [24–26]. In addition, only two seasons were analyzed. Consequently, it may be of interest for future studies to consider these limitations to further analyze match running and technical-tactical performance in professional soccer.

## CONCLUSIONS

Sports science, coaching, and medical staff may take the results from this study into consideration for developing training strategies. Regarding the technical-tactical performance, teams should focus on drills that enhance shooting accuracy and the total of shots performed in match play. However, defensive skills need to be reinforced considering the importance of goals conceded for team success. When it comes to match running performance, teams are encouraged to focus on offensive actions, in which they possess and run with the ball (especially at high speed) and defensive actions in which the players perform continuous and high-intensity physical efforts to prevent the opponents from scoring, avoid counterattacks, stay compact, and defend the area and goal. In this regard, strength and conditioning coaches need to train transitional play since running performance significantly increases during transitions [27]. Sprinting actions with and without ball possession are crucial and should be applied in training, not only by professional soccer players but also by players who aspire to be selected to play professionally. In this regard, medium- and large-sided games can be implemented to ensure high-speed running and sprinting exposure [28]. Moreover, players should be trained to perform at maximum intensity during the most demanding passages of play [29–31] while integrating a contextualized approach [32, 33].
